# Correction for Regueira-Iglesias et al., “Impact of 16S rRNA Gene Redundancy and Primer Pair Selection on the Quantification and Classification of Oral Microbiota in Next-Generation Sequencing”

**DOI:** 10.1128/spectrum.00043-24

**Published:** 2024-02-08

**Authors:** Alba Regueira-Iglesias, Lara Vázquez-González, Carlos Balsa-Castro, Triana Blanco-Pintos, Nicolás Vila-Blanco, Maria José Carreira, Inmaculada Tomás

## AUTHOR CORRECTION

Volume 11, no. 2, e04398-22, 2023, https://doi.org/10.1128/spectrum.04398-22. In the first sentence of the Acknowledgments section, “funded by Instituto de Salud Carlos III (ISCIII) through project PI21/00588 and cofunded by the European Union” should read “funded by Instituto de Salud Carlos III (ISCIII) through projects PI17/01722 and PI21/00588 and cofunded by the European Union (UE).”

