# Supplementary material for: Impact of 16S rRNA Gene Redundancy and Primer Pair Selection on the Quantification and Classification of Oral Microbiota in Next-Generation Sequencing
Source: Microbiol Spectr. 2023 Feb 13;11(2):e04398-22. doi: 10.1128/spectrum.04398-22 (PMC10101033; doi:10.1128/spectrum.04398-22)
Supplement: Supplemental file 1 — Legends to Tables S1 to S9. Download spectrum.04398-22-s0001.pdf, PDF file, 0.2 MB [file spectrum.04398-22-s0001.pdf]

SUPPLEMENTAL MATERIAL

Table S1. Supplemental file S1 contains tables with the sizes of the bacterial genomes and genes, the number of genes per genome, and the number of gene variants per genome across eight taxonomic ranks.

Table S2. Supplemental file S2 contains tables with the sizes of the archaeal genomes and genes, the number of genes per genome, and the number of gene variants per genome across eight taxonomic ranks.

Table S3. Supplemental file S3 contains tables with the species with and without matching amplicons and coverage estimators for all primer pairs tested.

Table S4. Supplemental file S4 contains tables with the overestimation factors of bacterial species using the bacterial-specific primer pairs.

Table S5. Supplemental file S5 contains tables with the overestimation factors of archaeal species using the archaeal-specific primer pairs.

Table S6. Supplemental file S6 contains tables with the overestimation factors of bacterial and archaeal species using the bacteria and archaea primer pairs.

Table S7. Supplemental file S7 contains a table with the bacterial and archaeal species with matching amplicons using the bacterial-specific and the archaeal-specific primer pairs, respectively.

Table S8. Supplemental file S8 contains a table with the bacterial and archaeal species with matching amplicons using the bacteria and archaea primer pairs.

Table S9. Supplemental file S9 contains tables with the NCBI taxonomy and identifiers of the oral-bacteria and the oral-archaea genomes.
